# Native sulfur/chlorine SAD phasing for serial femtosecond crystallography

**DOI:** 10.1107/S139900471501857X

**Published:** 2015-11-27

**Authors:** Takanori Nakane, Changyong Song, Mamoru Suzuki, Eriko Nango, Jun Kobayashi, Tetsuya Masuda, Shigeyuki Inoue, Eiichi Mizohata, Toru Nakatsu, Tomoyuki Tanaka, Rie Tanaka, Tatsuro Shimamura, Kensuke Tono, Yasumasa Joti, Takashi Kameshima, Takaki Hatsui, Makina Yabashi, Osamu Nureki, So Iwata, Michihiro Sugahara

**Affiliations:** a Department of Biological Sciences, Graduate School of Science, The University of Tokyo, 7-3-1 Hongo, Bunkyo-ku, Tokyo 113-0033, Japan; b RIKEN SPring-8 Center, 1-1-1 Kouto, Sayo-cho, Sayo-gun, Hyogo 679-5148, Japan; c Department of Physics, POSTECH, Pohang 790-784, Republic of Korea; d Institute for Protein Research, Osaka University, 3-2 Yamadaoka, Suita, Osaka 565-0871, Japan; e Division of Food Science and Biotechnology, Graduate School of Agriculture, Kyoto University, Gokasho, Uji, Kyoto 611-0011, Japan; f Department of Cell Biology and Anatomy, Graduate School of Medicine, The University of Tokyo, 7-3-1 Hongo, Bunkyo-ku, Tokyo 113-0033, Japan; g Department of Applied Chemistry, Graduate School of Engineering, Osaka University, 2-1 Yamadaoka, Suita, Osaka 565-0871, Japan; h Department of Structural Biology, Graduate School of Pharmaceutical Sciences, Kyoto University, 46-29 Yoshida Shimoadachi-cho, Sakyo-ku, Kyoto 606-8501, Japan; i Department of Cell Biology, Graduate School of Medicine, Kyoto University, Yoshidakonoe-cho, Sakyo-ku, Kyoto 606-8501, Japan; j Japan Synchrotron Radiation Research Institute, 1-1-1 Kouto, Sayo-cho, Sayo-gun, Hyogo 679-5198, Japan

**Keywords:** serial femtosecond crystallography, X-ray free-electron laser, sulfur/chlorine SAD

## Abstract

Serial femtosecond crystallography (SFX) allows structures to be determined with minimal radiation damage. However, phasing native crystals in SFX is not very common. Here, the structure determination of native lysozyme from single-wavelength anomalous diffraction (SAD) by utilizing the anomalous signal of sulfur and chlorine at a wavelength of 1.77 Å is successfully demonstrated. This sulfur SAD method can be applied to a wide range of proteins, which will improve the determination of native crystal structures.

## Abstract 2

Sulfur SAD phasing facilitates the structure determination of diverse native proteins using femtosecond X-rays from free-electron lasers *via* serial femtosecond crystallography.

## 1. Introduction   

Serial femtosecond crystallography (SFX) using ultrashort pulses from X-ray free-electron lasers (XFELs) has become an area of intense investigation. In particular, this method takes advantage of the acquisition of high-resolution protein structures by the ‘diffraction-before-destruction’ approach (Chapman *et al.*, 2011 ▸; Neutze *et al.*, 2000 ▸; Schlichting & Miao, 2012 ▸; Barty *et al.*, 2012 ▸; Emma *et al.*, 2010 ▸; Ishikawa *et al.*, 2012 ▸). SFX has successfully been used to collect data from small protein crystals on the micrometre to submicrometre scale (Boutet *et al.*, 2012 ▸; Barends, Foucar, Botha *et al.*, 2013 ▸; Johansson *et al.*, 2012 ▸; Redecke *et al.*, 2013 ▸; Liu, Wacker *et al.*, 2013 ▸; Weierstall *et al.*, 2014 ▸; Sugahara *et al.*, 2015 ▸) and has also been applied in time-resolved studies of light-driven structural changes and chemical dynamics (Kern *et al.*, 2014 ▸; Kupitz *et al.*, 2014 ▸; Tenboer *et al.*, 2014 ▸). The determined protein structures have largely been obtained using the molecular-replacement method (Chapman *et al.*, 2011 ▸; Boutet *et al.*, 2012 ▸; Johansson *et al.*, 2012 ▸; Redecke *et al.*, 2013 ▸; Liu, Wacker *et al.*, 2013 ▸; Weierstall *et al.*, 2014 ▸; Sugahara *et al.*, 2015 ▸; Kern *et al.*, 2014 ▸; Kupitz *et al.*, 2014 ▸; Tenboer *et al.*, 2014 ▸). Recently, gadolinium SAD phasing has successfully been demonstrated (Barends, Foucar, Botha *et al.*, 2013 ▸), but experimental phasing for routine structure determination still remains a challenge in SFX.

For the *de novo* phasing of macromolecules, experimental phasing has mainly been performed on heavy-atom derivatives of protein crystals, including the aforementioned Gd-SAD (Barends, Foucar, Botha *et al.*, 2013 ▸). However, the preparation of a heavy-atom derivative is usually a difficult task with unreliable results. In contrast, the SAD method using the anomalous signal arising from the ubiquitous S atoms in native proteins may be used to solve the crystallo­graphic phase problem, which is one of the limiting steps in the determination of macromolecular structure (Hendrickson & Teeter, 1981 ▸). Sulfur SAD uses native proteins, and does not require the painstaking procedure of producing heavy-atom derivatives. However, despite the advantage in sample preparation, SFX phasing with sulfur SAD has not yet been demonstrated because it requires a large degree of data multiplicity to utilize the relatively weak anomalous signal (Barends, Foucar, Shoeman *et al.*, 2013 ▸; Schlichting, 2015 ▸). Furthermore, it remains to be verified whether the Monte Carlo integration of partial Bragg reflections in SFX can accurately capture the weak anomalous differences in sulfur SAD phasing.

Here, we demonstrate the successful phasing of native proteins in SFX by applying sulfur/chlorine SAD phasing to determine the structure of lysozyme, with ten sulfur sites and one chloride. Native lysozyme crystals were used without any additional sample handling and a grease matrix was used as a protein carrier for serial sample loading to reduce sample consumption (Sugahara *et al.*, 2015 ▸).

## 2. Materials and methods   

### 2.1. Sample preparation   

Lysozyme crystals with a size distribution of between 7 and 10 µm were prepared following previously reported protocols (Sugahara *et al.*, 2015 ▸). The crystal number density was maintained at ∼7 × 10^7^ crystals per millilitre. The samples were stored at 4°C. In this study, we used a synthetic grease (Super Lube No. 21030, Synco Chemical Co.) as the crystal carrier matrix, with a lower background scattering compared with conventional mineral oil-based grease. The small crystals were mixed with grease using the procedure reported by Sugahara *et al.* (2015 ▸).

### 2.2. Single-shot data collection   

We carried out the experiments using femtosecond X-ray pulses from the SPring-8 Angstrom Compact Free Electron Laser (SACLA; Ishikawa *et al.*, 2012 ▸). The X-ray wavelength was kept at 1.77 Å (7 keV) with a pulse energy of ∼200 µJ. Each X-ray pulse delivers ∼7 × 10^10^ photons within a 10 fs duration (FWHM) to the samples with grease. Data were collected using X-ray beams of 1.5 × 1.5 µm focused by Kirkpatrick–Baez mirrors (Yumoto *et al.*, 2013 ▸). The crystals in the grease matrix were serially loaded using a syringe injector installed in a helium-ambiance diffraction chamber. The experiments were carried out using the Diverse Application Platform for Hard X-ray Diffraction in SACLA (DAPHNIS; Tono *et al.*, 2015 ▸) at BL3 (Tono *et al.*, 2013 ▸). The microcrystals embedded in grease were kept at a temperature of approximately 20 or 4°C. The sample chamber was kept at a temperature of ∼25°C and a humidity greater than 80%. One data set (data set C in Table 1 ▸) was collected using a new high-viscosity micro-extrusion injector system in air. Diffraction patterns were collected using a custom-built multiport CCD (Kameshima *et al.*, 2014 ▸). The grease matrix with randomly oriented crystals was extruded through injector nozzles with inner diameters of 50, 100 and 110 µm.

### 2.3. Structure determination   

Diffraction patterns were retrieved through the SACLA API (Joti *et al.*, 2015 ▸) and were filtered by *Cheetah* (Barty *et al.*, 2014 ▸). Each pattern with more than 20 spots was accepted as a hit and was indexed and integrated using *CrystFEL* (White *et al.*, 2012 ▸). Diffraction peak positions were determined using the built-in Zaefferer algorithm and passed on to *DirAx* (Duisenberg, 1992 ▸) for indexing. No sigma cutoff or saturation cutoff were applied. Measured diffraction intensities of each shot were scaled by the resolution-independent scale factor *k* before merging. Monte Carlo integrated intensities from *CrystFEL* were converted to XDS_ASCII format (Kabsch, 2010 ▸). Substructure search, phasing and phase improvement were carried out using *SHELXC*, *SHELXD* and *SHELXE* (Sheldrick, 2010 ▸). Prior knowledge of the correct structure was not used for phasing. The final *SHELXE* model was fed into *Buccaneer* (Cowtan, 2006 ▸) from the *CCP*4 suite (Winn *et al.*, 2011 ▸). Manual model revision and structure refinement were performed using *Coot* (Emsley & Cowtan, 2004 ▸) and *PHENIX* (Adams *et al.*, 2010 ▸), respectively. Details of the data-collection and refinement statistics are summarized in Tables 1 ▸ and 2 ▸.

## 3. Results   

### 3.1. Data collection   

**Figure 1:**
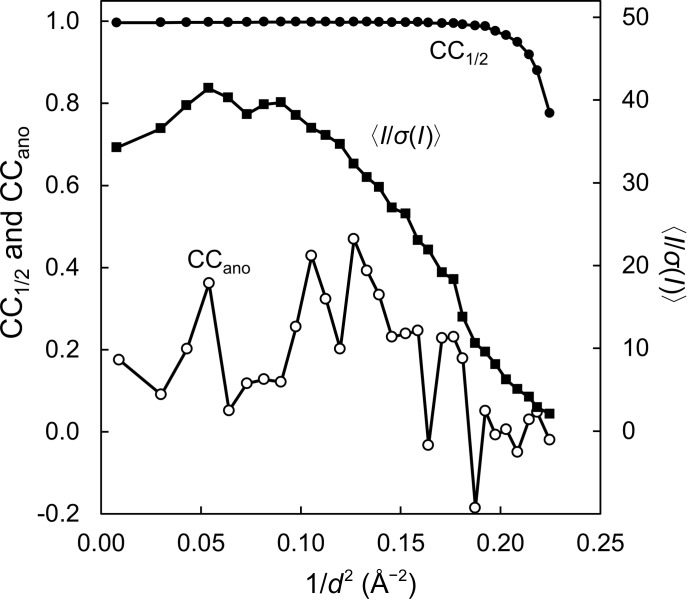
Quality of a data set of 150 000 patterns for sulfur SAD phasing. CC_1/2_ (black circle), CC_ano_ (empty circle) and *I*/σ(*I*) (black square) were plotted as a function of resolution.

With SACLA running at a 30 Hz repetition rate, we collected ∼700 000 diffraction patterns in ∼6 h. Data were collected under four different conditions of flow rate, injector nozzle size and temperature (Table 1 ▸). In total, about 200 µl of the sample volume was used with a crystal density of 6–7 × 10^7^ crystals per millilitre (Table 2 ▸). From approximately 450 000 hits, each with more than 20 diffraction spots, we successfully indexed and integrated approximately 180 000 patterns. As we show below, 150 000 indexed patterns were sufficient for sulfur SAD phasing of lysozyme crystals (space group *P*4_3_2_1_2). Microcrystals of 7–10 µm in size were used to acquire data sets at 2.1 Å resolution with a completeness of 100%, a CC_1/2_ of 0.998 and a CC_ano_ of 0.187 (Fig. 1 ▸). The overall 〈*I*/σ(*I*)〉 of the averaged observations was 24.9.

During Monte Carlo integration, the --scale option in the *process_hkl* program was essential for accurate SAD phasing. This simple linear scaling of intensities partially compensates for crystal size variations and fluctuations in each pulse intensity. Without scaling, substructure determination was not possible, even with 180 000 indexed patterns. Simply merging the unscaled intensities of 180 000 indexed patterns resulted in a poorer *d*′′/σ, which was comparable to that from a scaled data set with only 70 000 indexed patterns.

### 3.2. Phasing   

**Figure 2:**
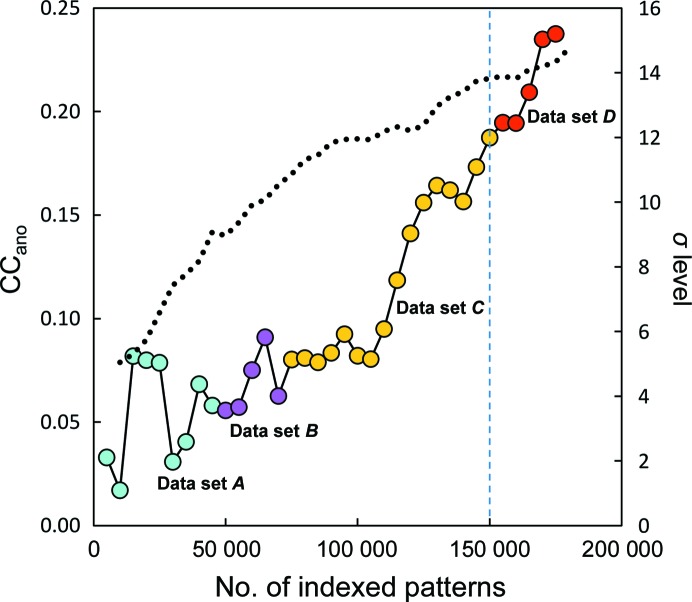
Quality of a merged data set of 180 000 patterns. CC_ano_ was plotted as a function of pattern number. Four data sets are shown as cyan (data set A), magenta (data set B), yellow (data set C) and red (data set D) circles. The black dotted line is a plot of the σ-level height of the Met105 S atom obtained from *ANODE*. A data set of 150 000 patterns (blue dashed line) was used for sulfur SAD phasing.

**Figure 3:**
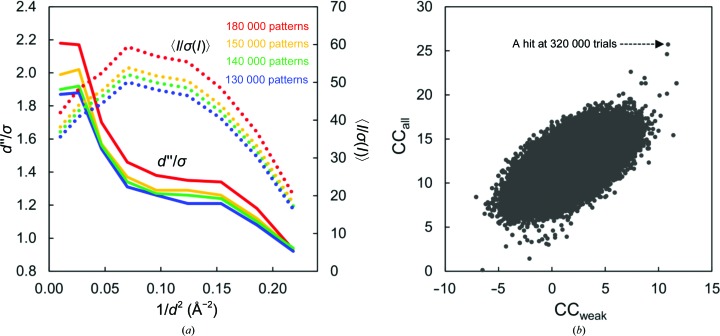
Results of *SHELXC* and *SHELXD*. (*a*) Statistics of the anomalous signal *d*′′/σ (solid line) and *I*/σ(*I*) (dotted line) produced by *SHELXC*. Four data sets of 180 000, 150 000, 140 000 and 130 000 patterns are coloured red, yellow, green and blue, respectively. (*b*) Correlation coefficient ratios (CC_all_/CC_weak_) from *SHELXD* using 150 000 patterns at 2.2 Å resolution.

Lysozyme is a 14 kDa protein consisting of 129 residues, including two methionines and eight cysteines forming four disulfide bridges. The expected magnitude of the anomalous signal is estimated by the formula (Hendrickson & Teeter, 1981 ▸; Dauter *et al.*, 1999 ▸)
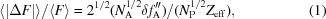
where *N*_A_ is the number of anomalous scatterers (ten S atoms with a δ*f*′′ value of 0.72), *N*_P_ is the number of protein atoms (1001 atoms) and *Z*_eff_ is the average scattering of protein atoms (6.7 electrons). Thus, the estimated anomalous difference is about 1.5%. We obtained four data sets (A–D in Table 1 ▸) at different beam times using different crystallization batches at 2.1 Å resolution with a completeness of 100%, an *R*_split_ ranging from 4.1 to 6.6% and a Wilson *B* factor ranging from 42.7 to 52.6 Å^2^. There were no significant differences among the four data sets. The correlation coefficients among the four data sets (A–D) were 0.99. On the other hand, the CC_ano_ values were rather poor, varying from 0.002 to 0.136, owing to the small number of indexed patterns in each subset. The crystal structure was solved using merged data from the four data sets (Fig. 2 ▸). Using 180 000, 150 000, 140 000 and 130 000 patterns, we estimated the *d*′′/σ from *SHELXC*, indicating anomalous signal (Fig. 3 ▸ *a*). Substructure determination and phasing were performed by *SHELXD* and *SHELXE* (Sheldrick, 2010 ▸).

Firstly, substructure determination was performed in *SHELXD* with various high-resolution limits (2.0–3.0 Å in 0.1 Å steps, with up to 500 000 trials per condition). 500 000 trials in *SHELXD* took 4 h on a 32-core machine (Fig. 3 ▸ *b*). For a data set merged from 150 000 patterns, the best solution was found after about 320 000 trials using reflections to 2.2 Å resolution. With more indexed patterns, correct solutions could be found more easily, in less than 5000 *SHELXD* trials at lower resolution. For example, when 180 000 indexed patterns were merged, we could solve the substructure at 2.7 Å resolution but not at 2.8 Å. We succeeded in locating the S^γ^ atoms of Cys6, Cys30, Cys76, Cys80, Cys94 and Cys115 (but not Cys64 and Cys127) and the S^δ^ atoms of Met12 and Met105 as well as one chloride ion from the harvest buffer [10%(*w*/*v*) sodium chloride, 0.1 *M* sodium acetate pH 3.0]. Correlation coefficients at 2.2 Å resolution cutoff were 25.7 for all data. There was a steep decrease in occupancy after the ninth site. The *B* factors for Cys64 and Cys127 were not significantly higher than for other S atoms (Table 3 ▸). Cys64 and Cys127 form disulfide bonds with Cys80 and Cys6, respectively, which makes it difficult to separate their peaks from those of their neighbours.

Next, *SHELXE* runs were submitted for substructure refinement and phase calculation. Combinations of the solvent content (-s option; 44–50% in 1% steps), the number of substructure atoms employed (-h and -z options; nine, ten and 11 sites) and the high-resolution cutoff for calculation (-d option; 2.1, 2.2 and 2.3 Å) were systemically tested. 25 cycles of automatic chain tracing were carried out (-a option). The results were compared using the CC of the partial structure against the native data. For the 150 000 pattern data set, using 11 sites, a solvent content of 44% and a high-resolution limit at 2.1 Å, *SHELXE* traced 90 of 129 residues. Although the occupancies of the tenth and eleventh sites were low, their inclusion improved the result.

**Figure 4:**
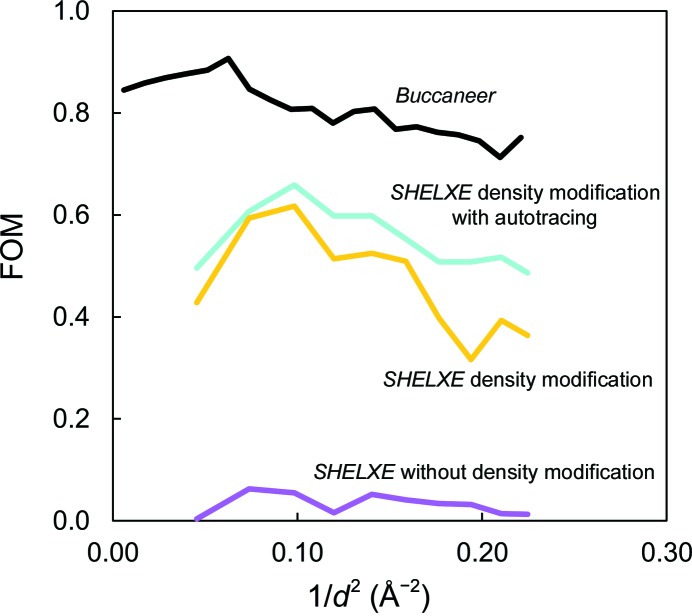
Quality of the SAD phases. The figure of merit (FOM) from *SHELXE* without density modification (magenta line), *SHELXE* with density modification (yellow line), *SHELXE* with density modification and autotracing (cyan line) and *Buccaneer* (black line) are plotted as a function of resolution.

**Figure 6:**
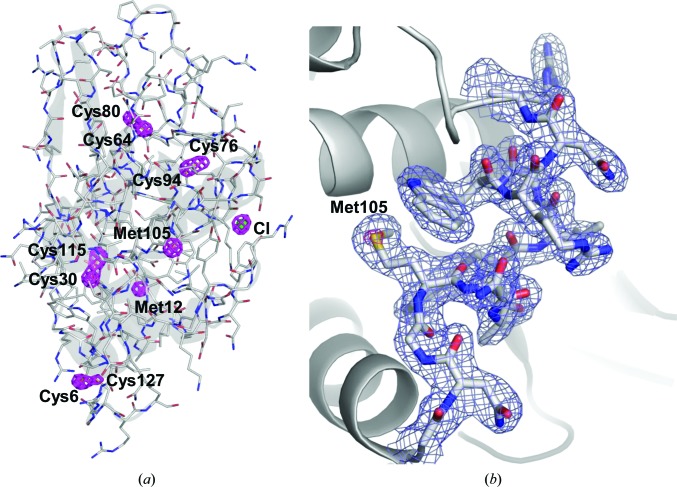
Experimental phasing of lysozyme. (*a*) Stick model of the refined lysozyme model superimposed onto the anomalous difference Fourier map calculated by *ANODE*, showing the sulfur and chlorine sites (coloured magenta). Bound chloride ion is depicted as a green sphere. The map contoured at the 6.0σ level. (*b*) A close-up view of the lysozyme structure with a 2*F*_o_ − *F*_c_ electron-density map contoured at the 1.0σ level (coloured blue). An anomalous difference Fourier map contoured at the 10.0σ level shows the S atom of Met105 (coloured pink). These figures were drawn with *PyMOL* (http://www.pymol.org).

Subsequently, 100% of the structure was constructed automatically with side chains by *Buccaneer* (Cowtan, 2006 ▸). Finally, we refined the structure to an *R* and *R*_free_ of 15.7 and 19.1%, respectively (PDB entry 4yop). The phase qualities and the electron-density maps at each stage in the phasing process are shown in Figs. 4 ▸ and 5 ▸, respectively. The final anomalous difference Fourier map in Fig. 6 ▸, which was generated by *ANODE* (Thorn & Sheldrick, 2011 ▸), displays significant anomalous peak heights (14–8σ level) for the 11 atoms that were located (Table 3 ▸).

## 4. Discussion   

Using synchrotron radiation, Dauter and coworkers showed that small anomalous differences can be used to solve the phase problem (Dauter *et al.*, 1999 ▸). They utilized the anomalous signal from S and Cl atoms in a lysozyme crystal measured at a wavelength of 1.54 Å (the anomalous scattering factor *f*′′ of the S atom is 0.56). In SFX, we injected ∼200 µl of crystal suspension at 10^7^ crystals per millilitre. Each crystal measured around 10 × 7 × 7 µm in size. Thus, the total volume of the crystals used was about 9.8 × 10^8^ µm^3^. To obtain this quantity, we used ∼5 mg of lysozyme. On the other hand, Dauter *et al.* (1999 ▸) collected a complete data set from a single crystal of 300 × 300 × 400 µm in size; that is, 3.6 × 10^7^ µm^3^. This is 27 times smaller than the total volume of the crystals used in the SFX study. Although they used 2 ml batches of a 20 mg ml^−1^ protein solution, a single crystal of similar size could have been obtained from a single drop of crystallization solution on a vapour-diffusion plate. Indeed, recent reports of sulfur SAD have mostly used crystals obtained by vapour diffusion, which typically requires only a few micrograms of protein. Thus, the current SFX method is inefficient in terms of sample consumption. Native SAD phasing at XFELs requires a larger volume of crystals and many hours of beam time. However, some targets (for example, the rhodopsin–arrestin complex; Kang *et al.* 2015 ▸) yield crystals that are only measurable in the SFX regime. Although all of these targets have been phased by molecular replacement, it is likely that new targets will be identified in the near future that cannot be phased by molecular replacement. Our results demonstrate that these targets can be solved even if heavy-atom derivatization is unsuccessful.

Synchrotron-based serial crystallography data collection has been attempted and anomalous signal from S atoms in lysozyme has been detected but lacked sufficient phasing power to obtain an interpretable electron-density map (Botha *et al.*, 2015 ▸). In this study, SAD phasing was successfully demonstrated using femtosecond X-ray pulses at 1.77 Å wavelength using 150 000 indexed patterns to 2.1 Å resolution. In a previous study, Barends, Foucar, Shoeman *et al.* (2013 ▸) detected anomalous signal from sulfur in SFX at 1.7 Å wavelength using 43 840 indexed patterns to 3.2 Å resolution; however, it was insufficient for phasing. We could not solve the structure when our data set was truncated to the same number of indexed patterns and resolution, indicating that a highly redundant data at a resolution beyond 2.7 Å is required for native SAD phasing.

Collecting data at longer wavelengths is a powerful technique to solve the crystallographic phase problem by exploiting the small anomalous signals from sulfur or phosphorus present in native protein or RNA/DNA crystals. These anomalous signals are also useful to identify many biologically important light atoms and ions, including chloride, as shown in this work, calcium (*e.g.* the photosystem II protein complex; Ferreira *et al.*, 2004 ▸) and potassium. Currently, wavelengths of 1.7 Å (sulfur *f*′′ = 0.67) or 1.9–2.7 Å (sulfur *f*′′ = 0.82–1.52) are commonly used for sulfur SAD structure determination (Rose *et al.*, 2015 ▸). Robust procedures have been reported for enhancing the signal-to-noise ratio in diffraction measurements by combining data sets from multiple crystals (Liu *et al.*, 2012 ▸; Liu, Liu *et al.*, 2013 ▸). This has successfully been applied to flavivirus nonstructural protein 1 (Akey *et al.*, 2014 ▸). However, a number of practical problems still remain; for example, on the I23 beamline at Diamond Light Source, cooled crystals are introduced into a vacuum using a robot (Mykhaylyk & Wagner, 2013 ▸). This is essential in order to avoid air absorption/scattering and radiation-damage problems. Here, it is also necessary to address a major source of systematic error in diffraction data: that is, the absorption of diffracted X-rays by the crystal sample itself and its mount.

SFX has the potential to solve most of these problems. It has already been shown that the sample injector used for SFX experiments is compatible with vacuum (Chapman *et al.*, 2011 ▸) and helium (Tono *et al.*, 2015 ▸) chambers. In addition, our paper shows that we can collect accurate anomalous signals at a long wavelength with SFX. Here, the data did not suffer from radiation damage, and crystal irregularities were compensated for by using many crystals in different orientations. This study demonstrates the potential of SFX for long-wavelength macromolecular crystallography. In particular, it is noteworthy that our sulfur SAD phasing was performed successfully even in the presence of a ring-shaped background owing to scattering from grease (Supplementary Fig. S1). Currently, we are studying crystal carrier matrices with a low background scattering that are suitable for long-wavelength experiments.

We have successfully applied native sulfur/chlorine SAD phasing to SFX for the first time; however, a report of another sulfur SAD phasing experiment was presented and discussed by Nass and coworkers at the Second International BioXFEL Conference (unpublished work). This is a major breakthrough for native phasing of radiation-sensitive proteins in macromolecular crystallography, which complements the conventional rotation/oscillation-based methods at synchrotrons. Future XFELs with higher repetition rates will certainly make data collection at high multiplicity and low signal-to-noise ratios easier and quicker. Developments in data-processing algorithms, especially post-refinement, will improve data quality and reduce the number of necessary frames. These advancements will make sulfur SAD phasing with SFX more accessible to wider range of samples.

## Supplementary Material

PDB reference: lysozyme, 4yop

Supplementary Figure S1.. DOI: 10.1107/S139900471501857X/wa5097sup1.pdf

## Figures and Tables

**Figure 5:**
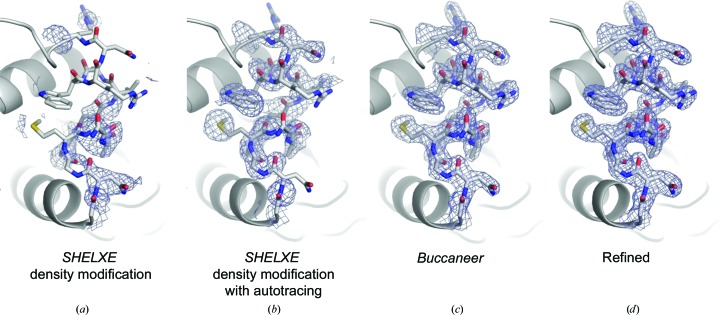
2*F*_o_ − *F*_c_ electron-density maps contoured at the 1.0σ level from the various steps of the phasing process. SAD phasing was performed by (*a*) *SHELXE* with density modification, (*b*) *SHELXE* with density modification and autotracing and (*c*) *Buccaneer* with automatic model building; (*d*) shows the final refined map. These figures were drawn with *PyMOL* (http://www.pymol.org).

**Table 1:** Experimental conditions and crystallographic statistics Values in parentheses are for the outermost shell.

Data set	A	B	C	D
Nozzle ID (µm)	50	110	100	110
Flow rate (µl min^−1^)	0.17	0.48	0.96	0.48
Temperature (°C)	4	20	20	20
Space group	*P*4_3_2_1_2			
Unit-cell parameters				
*a* (Å)	79.1	78.6	80.2	79.5
*b* (Å)	79.1	78.6	80.2	79.5
*c* (Å)	37.9	37.8	38.5	38.2
No. of collected images	190839	99055	299202	99747
No. of hits	83804	44581	269923	54465
No. of indexed patterns	44522	27670	78164	29218
Indexing rate from hits (%)	53.1	62.1	29.0	53.6
Total No. of reflections	4893481	2982074	8995984	3174292
No. of unique reflections	13420	13244	14049	13699
Resolution range (Å)	40–2.1 (2.12–2.10)	40–2.1 (2.12–2.10)	40–2.1 (2.12–2.10)	40–2.1 (2.12–2.10)
Completeness (%)	100 (100)	100 (100)	100 (100)	100 (100)
*R*_split_ † (%)	6.0 (26.0)	6.6 (37.6)	4.1 (26.9)	6.4 (27.6)
CC_1/2_	0.993 (0.905)	0.993 (0.786)	0.997 (0.898)	0.992 (0.885)
CC_ano_	0.002 (−0.035)	0.117 (−0.075)	0.136 (−0.138)	0.076 (−0.041)
〈*I*/*σ*(*I*)〉	14.8 (3.63)	11.9 (2.70)	20.6 (3.64)	13.3 (3.54)
Wilson *B* (Å^2^)	42.7	48.6	52.6	50.2

† *R*_split_ = (1/2^1/2^)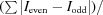 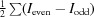.

**Table 2:** Crystallographic statistics Values in parentheses are for the outermost shell.

	Data sets A + B + C + D†
Data collection	
Wavelength (Å)	1.77
Space group	*P*4_3_2_1_2
Unit-cell parameters	
*a* (Å)	79.2
*b* (Å)	79.2
*c* (Å)	37.9
No. of collected images	688843
No. of hits	452773
No. of indexed patterns	179574
Indexing rate from hits (%)	39.7
No. of indexed patterns used	150000
Total No. of reflections	17379562
No. of unique reflections	15532
Resolution range (Å)	40–2.1 (2.12–2.10)
Completeness (%)	100 (100)
*R*_split_ ‡ (%)	3.1 (49.3)
CC_1/2_	0.998 (0.776)
CC_ano_	0.187 (−0.02)
〈*I*/σ(*I*)〉	24.9 (2.1)
Refinement	
*R*/*R*_free_ (%)	15.7/19.1
R.m.s. deviations	
Bond lengths (Å)	0.008
Bond angles (°)	1.074
Wilson *B* (Å^2^)	41.0
No. of atoms	
Protein	1000
Water	42
Chloride ion	1
Sodium ion	1
Mean *B* factor (Å^2^)	
Protein	41.0
Water	44.8
Chloride ion	41.64
Sodium ion	45.73
PDB code	4yop

† Details are shown in Table 1 ▸.

‡ *R*_split_ = (1/2^1/2^)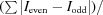 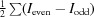.

**Table 3:** Peak heights of S and Cl atoms obtained from *ANODE*

	Fractional coordinates					
Peak	*x*	*y*	*z*	Height (σ)	Nearest residue	*B* factor (Å^2^)
1	0.347	−0.065	0.482	14.1	Met105	36.28
2	0.208	−0.010	0.283	12.1	Cys94	36.73
3	0.181	0.073	0.299	12.0	Cys64	33.34
4	0.163	0.094	0.301	11.6	Cys80	33.78
5	0.206	−0.014	0.264	11.6	Cys76	37.40
6	0.378	−0.007	0.638	11.5	Cys115	32.73
7	0.402	−0.104	0.314	11.4	Cl	41.64
8	0.256	−0.077	0.627	11.0	Met12	38.59
9	0.374	−0.020	0.689	10.9	Cys30	32.23
10	0.266	−0.107	0.923	10.6	Cys6	43.93
11	0.275	−0.115	0.905	8.0	Cys127	42.75
