# Supplementary material for: Native sulfur/chlorine SAD phasing for serial femtosecond crystallography
Source: Acta Crystallogr D Biol Crystallogr. 2015 Nov 27;71(Pt 12):2519–25. doi: 10.1107/S139900471501857X (PMC4667287; doi:10.1107/S139900471501857X)
Supplement: Supplementary file 1 [file d-71-02519-sup1.pdf]

BIOLOGICAL CRYSTALLOGRAPHY

Volume 71 (2015)

Supporting information for article:

Native sulfur/chlorine SAD phasing for serial femtosecond crystallography

Takanori Nakane, Changyong Song, Mamoru Suzuki, Eriko Nango, Jun Kobayashi, Tetsuya Masuda, Shigeyuki Inoue, Eiichi Mizohata, Toru Nakatsu, Tomoyuki Tanaka, Rie Tanaka, Tatsuro Shimamura, Kensuke Tono, Yasumasa Joti, Takashi Kameshima, Takaki Hatsui, Makina Yabashi, Osamu Nureki, So Iwata and Michihiro Sugahara

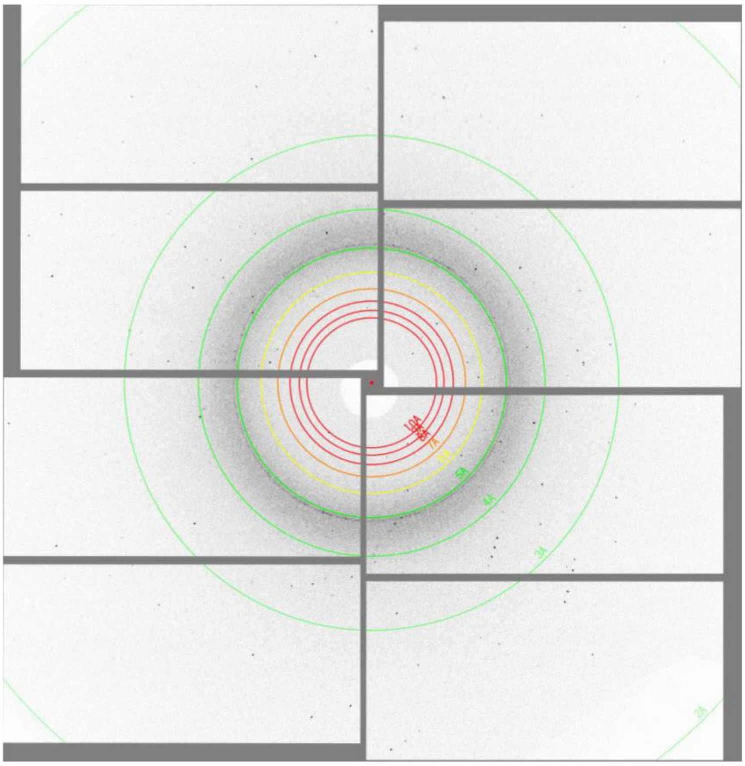

Figure S1 A typical XFEL single diffraction pattern from an individual lysozyme microcrystal in grease. The grease produces a background scattering of five to ten photons at 4 to 5 Å. The typical background level in other areas of the detector is less than one photon.
